# Multimodality Imaging for Diagnosing Transthyretin Cardiac Amyloidosis

**DOI:** 10.7759/cureus.37838

**Published:** 2023-04-19

**Authors:** Fathima Fijula Palot Manzil, Tarun Pandey

**Affiliations:** 1 Radiology/Nuclear Medicine, University of Arkansas for Medical Sciences, Little Rock, USA; 2 Radiology, University of Arkansas for Medical Sciences, Little Rock, USA

**Keywords:** noninvasive techniques, cardiac magnetic resonance imaging, pyp scintigraphy, transthyretin, cardiac amyloidosis

## Abstract

Amyloidosis is the result of the extracellular deposition of amyloid in various organs. Common types are light-chain and transthyretin amyloidosis. Cardiac amyloidosis (CA) is a restrictive cardiomyopathy caused by amyloid infiltration in cardiac tissues. The detection of CA is increasing with the advent of easily accessible imaging modalities. Early diagnosis ensures a better prognosis. We present a case of cardiac amyloidosis, diagnosed from specific imaging findings on cardiac magnetic resonance imaging and more precisely as transthyretin type based on findings on nuclear scintigraphy.

## Introduction

Amyloidosis is caused by the building up of proteins called amyloid in different organs. Endomyocardial biopsy is the definitive diagnostic modality of cardiac amyloidosis (CA) [1]. However, the noninvasive techniques improve patient acceptance of work-up to diagnose CA. Echocardiography provides clues like increased left ventricular thickness, prompting further investigations [2]. Strain imaging through echocardiography could be useful to know the cause of left ventricular hypertrophy. The relative apical sparing pattern of longitudinal strain distinguishes cardiac amyloidosis with good sensitivity and specificity. Cardiac magnetic resonance imaging (CMR) though has diagnostic accuracy for CA, has comparable findings like myocardial enhancement in other infiltrative cardiomyopathies as well. Technetium-99m pyrophosphate (99mTc-PYP) scintigraphy is a game changer, a positive study being specific for transthyretin cardiac amyloidosis (ATTR-CA) in the context of a negative monoclonal light chain screen [3].

## Case presentation

A 63-year-old 6 ft, 101 kg male with a long-standing history of dyspnea, hypertension, atrial fibrillation, and gout presented with worsening of shortness of breath, fatigue, and lower extremities edema concerning heart failure. Patient was on apixaban 2.5 mg, furosemide 40 mg, lisinopril 2.5 mg and bumetanide 2 mg.

On echocardiography, the left ventricle was normal in size (4.2 cm). The ejection fraction was estimated to be 40% and there was diffuse moderate hypokinesis. Wall thickness was moderate to markedly increased. There was moderate concentric hypertrophy. Diastolic features were consistent with a pseudonormal left ventricular filling pattern, with concomitant abnormal relaxation and increased filling pressure suggestive of grade 2 diastolic dysfunction. Right ventricle size and systolic function were normal. There was mild mitral regurgitation and the left atrium was markedly dilated. The transaortic, trans pulmonic, and trans tricuspid velocities were within the normal range with no evidence of stenosis or regurgitation. The tricuspid jet envelope definition was inadequate for the estimation of RV systolic pressure. The inferior vena cava was normal in size and respirophasic changes were normal. There was no pericardial effusion. The left atrial diameter was 4.5 cm, interventricular septal diameter 1.7 cm, left ventricular internal end-diastolic diameter 4.4 cm, left ventricular internal diameter end systole 3.1 cm, and left ventricular posterior wall end diastole 1.7 cm. There was markedly reduced peak global longitudinal strain at - 5.8% with a homogeneous reduction in deformation across all left ventricular myocardial segments with apical sparring.

Serum and urine electrophoresis were negative for monoclonal immunoglobulin. Urine electrophoresis showed proteins < 5 mg/dl. Specific M-protein could not be identified on serum immunofixation. Serum immunoglobulin kappa free light chain was 1.91 (normal: 0.33- 1.94 mg/dl), lambda free light chain 2.59 (normal: 0.57- 2.63 mg/dl), and K/L ratio 0.74 (normal: 0.26- 1.65). The patient was consulted by hematology and a bone marrow biopsy demonstrated normocellular marrow with trilineage hematopoiesis and no increased plasma cells.

**Figure 1:**
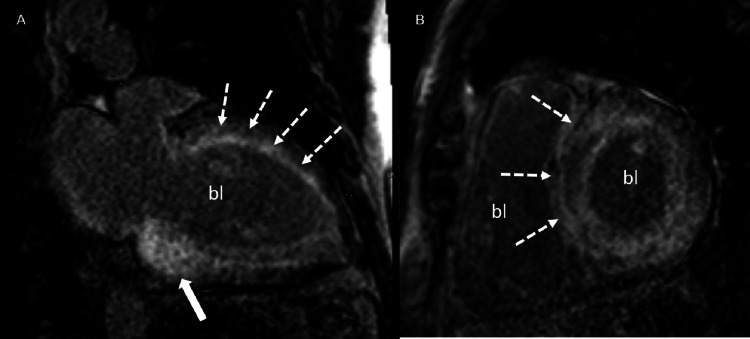
Cardiac magnetic resonance imaging Vertical long-axis (A) and short-axis (B) phase-sensitive post-contrast inversion recovery images demonstrate nonterritorial diffuse subendocardial enhancement (dotted arrows) characteristics of CA. Notice the loss of blood pool signal (bl) due to high circulating levels of amyloid. Note the somewhat confluent and prominent involvement at the base (solid arrow) and relative sparing of the left ventricular apex, another feature of CA.

**Figure 2:**
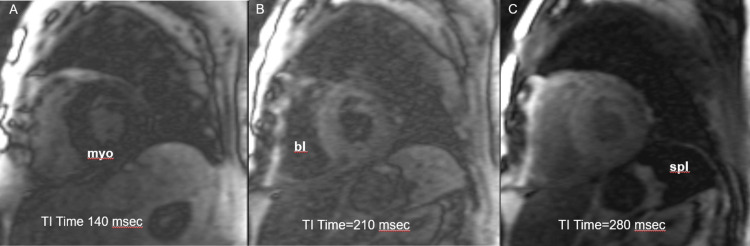
Cardiac magnetic resonance imaging Delayed post-contrast TI scout images show abnormal inversion times with a Type IV nulling pattern. The myocardium (myo) nulls before the blood pool at 140ms (A), the blood pool (bl) nulls later at 210ms (B) and splenic nulling (spl) occurs at 280 ms without coinciding with myocardial nulling (C).

CMR showed characteristic CA findings as explained in the figure legends (Figures 1, 2).

**Figure 3:**
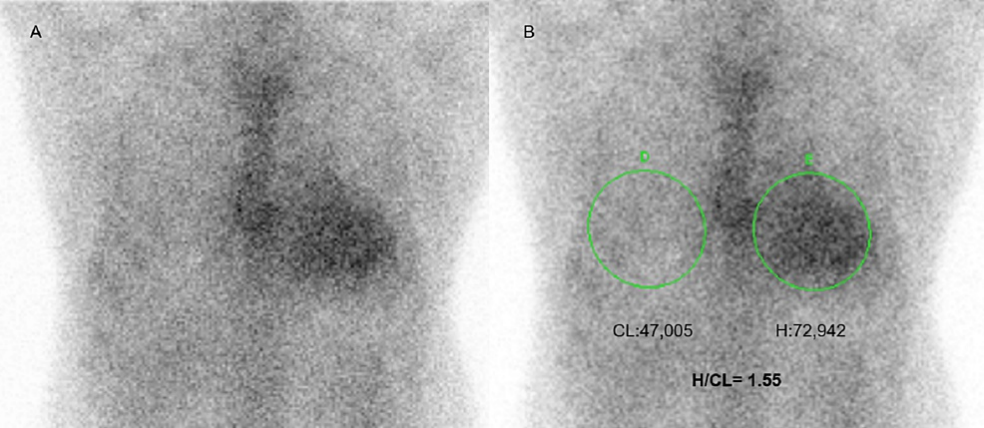
Technetium-99m pyrophosphate scintigraphy- planar imaging Pyrophosphate scintigraphy in anterior projection shows myocardial uptake more than rib (A). On quantitative analysis, H/CL is 1.55 (B).

The scintigraphy acquired one hour after an injection of 795.5 MegaBequerel (21.5 milliCurie) 99mTc-PYP showed specific features as described in the figure legends (Figure 3).

**Figure 4:**
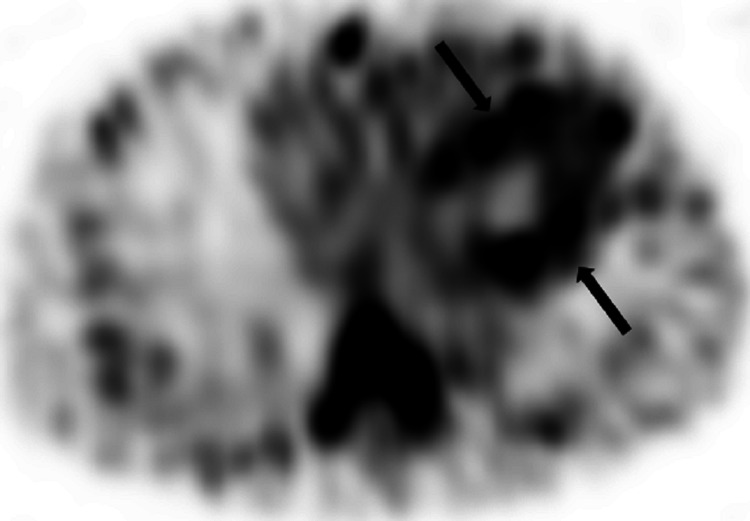
Technetium-99m pyrophosphate scintigraphy- SPECT SPECT of the chest shows uptake in the myocardium (arrows).

Single photon emission computed tomography (SPECT) confirmed the uptake is in the myocardium, not the blood pool (Figure 4).

Positive CMR and pyrophosphate (PYP) imaging with negative light chains were highly suggestive of ATTR-CA. ATTR-CA is further classified as wild-type ATTR (ATTRwt) and hereditary or variant-type ATTR (ATTRv) based on the TTR gene sequence [4]. Although TTR genetic testing is recommended for patients with an established diagnosis of ATTR-CA to differentiate between ATTRwt and ATTRv, it was not done in our patient due to patient unwillingness. The patient is considered for tafamidis, a transthyretin kinetic stabilizer.

## Discussion

Echocardiographic findings which provide clues to the diagnosis of cardiac amyloidosis include increased left ventricular thickness, grade 2 or more diastolic dysfunction, and decreased left ventricular global longitudinal strain. Characteristic cardiac magnetic resonance imaging findings include diffuse subendocardial or transmural late gadolinium enhancement and abnormal gadolinium kinetics [5,6].

On delayed post-contrast T1 scout images of CMR, the normal temporal nulling (losing signal) is classified as type 1 which follows an orderly pattern. Abnormal nulling patterns are types 2, 3, or 4, associated with a higher risk of CA (Table 1) [7]. In addition to MRI, especially in the developing and resource constraint setting, speckle tracking echocardiography and estimation of global longitudinal strain (GLS) can be beneficial in identifying CA [8]. Left ventricular longitudinal strain detected by two-dimensional speckle tracking echocardiography can point to myocardial function alterations at the early stages of the disease. 2D speckle tracking echocardiography consists of capturing and tracking of speckles along the cardiac cycle, generating motion vectors and deformation curves. It detects myocardial deformation and the percentage of deformation.

**Table 1:** Classification of nulling patterns on delayed post-contrast T1 scout cardiac magnetic resonance imaging

Classification of nulling patterns on delayed post-contrast T1 scout cardiac magnetic resonance imaging	
Type 1	Normal temporal nulling. Follows an orderly pattern of blood pool nulling, followed by myocardial nulling which coincides with splenic nulling
Type 2	Myocardial nulling preceding or coinciding with the blood pool nulling
Type 3	Myocardial nulling non-coincident with the splenic nulling
Type 4	If both types 2 and 3 seen

In the presence of typical findings on echocardiography and CMR and with the absence of histology and absent monoclonal gammopathy, a Tc-99m PYP scan can help to further evaluate for ATTR-CA. PYP scintigraphy is acquired in anterior projection one-hour post-injection of the tracer. A qualitative visual evaluation comparing cardiac to rib uptake is graded as grades 0, 1, 2, and 3 (Table 2) [9]. Scores ≥ 2 are ATTR positive if clonal dyscrasias are excluded. Quantitative evaluation is done by drawing similar regions of interest over the heart and contralateral chest and calculating the count ratio. Heart to contralateral (H/CL) count ratio ≥ 1.5 is suggestive of ATTR. Lateral and left anterior oblique images could be obtained for confirmation. If there is persistent blood pool activity at 1 hour, imaging should be repeated at three hours. Causes of false positive scintigraphy include light-chain CA, ApoA1 CA, myocardial infarction, rib fracture, and blood pool activity. SPECT can additionally evaluate the tracer distribution. 99mTc-DPD and 99mTc-HMDP are other radioligands for CA imaging [10,11]. 

**Table 2:** Qualitative visual scoring for transthyretin cardiac amyloidosis on PYP scintigraphy

Qualitative visual scoring for transthyretin cardiac amyloidosis on PYP scintigraphy	
Grade 0	No myocardial uptake
Grade 1	Myocardial uptake < rib uptake
Grade 2	Myocardial uptake = rib uptake
Grade 3	Myocardial uptake > rib uptake

## Conclusions

Cardiac amyloidosis is a major cause of morbidity and mortality. If histological confirmation is not feasible and there are predictable findings of amyloidosis on echocardiogram and CMR, ATTR-CA is diagnosed by characteristic patterns on PYP scan when monoclonal gammopathy is excluded. Cardiac or extracardiac biopsy with amyloid typing is necessary when monoclonal protein is present. Cardiac transplantation does benefit some patients. Emerging therapies that inhibit amyloidogenic misfolding of TTR are promising. Our patient had typical representative findings of CA on CMR and 99mTc-PYP scan and is now considered for therapy with a transthyretin kinetic stabilizer.
